# Vkorc1 gene polymorphisms confer resistance to anticoagulant rodenticide in Turkish rats

**DOI:** 10.7717/peerj.15055

**Published:** 2023-05-02

**Authors:** Nuri Yiğit, Mustafa T. Duman, Derya Çetintürk, Fulya Saygılı-Yiğit, Ercüment Çolak, Reyhan Çolak

**Affiliations:** 1 Biology Department/Faculty of Science, Ankara University, Ankara, Turkey; 2 Biotechnology Department/Faculty of Arts and Sciences, Niğde Ömer Halisdemir University, Niğde, Turkey

**Keywords:** Vkorc1, Anticoagulant resistance, Mutation, Black rat, Brown rat

## Abstract

Mutations in Exon 1, 2 and 3 of the vitamin K epoxide reductase complex subunit 1 (*Vkorc1*) gene are known to lead to anticoagulant rodenticide resistance. In order to investigate their putative resistance in rodenticides, we studied the genetic profile of the *Vkorc1* gene in Turkish black rats (*Rattus rattus*) and brown rats (*Rattus norvegicus*). In this context, previously recorded Ala21Thr mutation (*R. rattus*) in Exon 1 region, Ile90Leu mutation (*R. rattus, R. norvegicus*) in Exon 2 region and Leu120Gln mutation (*R. norvegicus*) in Exon 3 region were identified as “missense mutations” causing amino acid changes. Ala21Thr mutation was first detected in one specimen of Turkish black rat despite the uncertainty of its relevance to resistance. Ile90Leu mutation accepted as neutral variant was detected in most of black rat specimens. Leu120Gln mutation related to anticoagulant rodenticide resistance was found in only one brown rat specimen. Furthermore, Ser74Asn, Gln77Pro (black rat) and Ser79Pro (brown rat) mutations that cause amino acid changes in the Exon 2 region but unclear whether they cause resistance were identified. In addition, “silent mutations” which do not cause amino acid changes were also defined; these mutations were Arg12Arg mutation in Exon 1 region, His68His, Ser81Ser, Ile82Ile and Leu94Leu mutations in Exon 2 region and Ile107Ile, Thr137Thr, Ala143Ala and Gln152Gln mutations in Exon 3 region. These silent mutations were found in both species except for Ser81Ser which was determined in only brown rats*.*

## Introduction

First- and second-generation anticoagulant rodenticides (FGARs, SGARs) are used to control these rodents, especially in urban areas in Turkey; however, it is unclear whether rats in Turkey may develop resistance to such rodenticides. Recent studies have shown that anticoagulant rodenticide resistance in rats is associated with the vitamin K epoxide reductase complex subunit 1 (*Vkorc1)* gene (Pelz et al., 2005; Buckle, 2013). The *Vkor* family includes enzymes that are present in vertebrates, plants, bacteria and archaea. *Vkorc1* encodes an integral membrane protein that catalyses the reduction of vitamin K 2,3-epoxide and vitamin K to vitamin K hydroquinone. Anticoagulant rodenticides bind to vitamin K epoxide reductase by targeting the warfarin-sensitive subcomponent encoded by the *Vkorc1* gene (Rost et al., 2004; Pelz et al., 2005; Ishizuka et al., 2008). In rats, the *Vkorc1* gene is located on chromosome 1 (Grandemange et al., 2009) andthe gene encodes a 161 amino acid protein product (Wang et al., 2008). Mutations in the exons of *Vkorc1* gene were found to have significant impact on the anticoagulant efficacy in brown rats (Meerburg et al., 2014; Goulois et al., 2016; Cowan et al., 2017; Iacucci et al., 2018; Boitet et al., 2017; Díaz & Kohn, 2021), buff-breasted rats (*Rattus flavipectus*) (Huang et al., 2011), black rats (Tanaka et al., 2012; Goulois et al., 2016; Díaz & Kohn, 2021) and kiore rats (*R. exulans*) (Cowan et al., 2017) in several regions around the globe.

In this study, sequences of the Exon 1, 2 and 3 coding regions of the *Vkorc1* gene were amplified and compared in Turkish brown and black rats. Single Nucleotide Polymophisms (SNPs) were then detected in order to investigate if possible mutations in these two species.

## Materials & Methods

**Figure 1:**
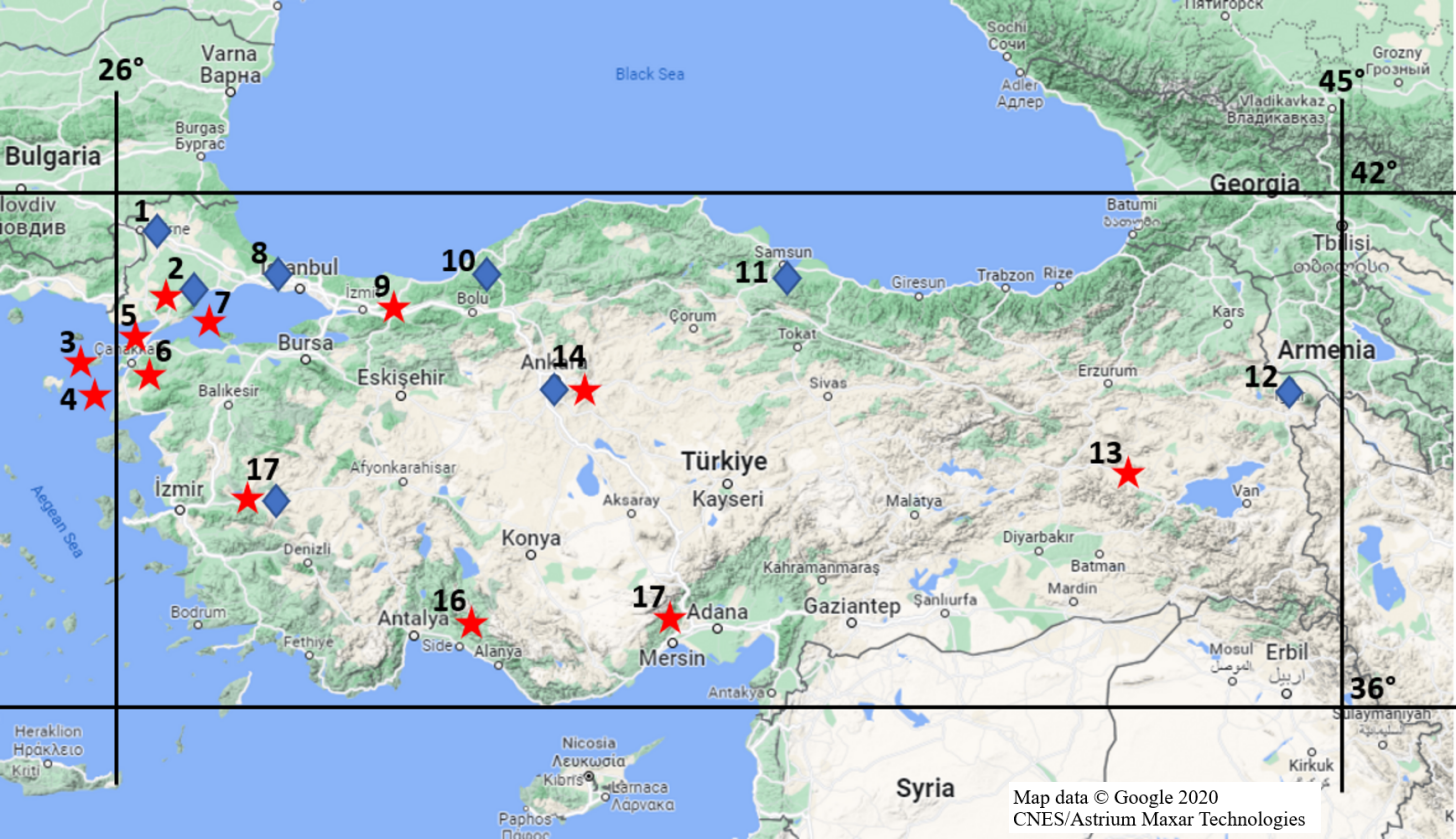
Provinces where rat specimens collected from in Turkey. Stars imply *R. rattus* specimens and diamonds show *R. norvegicus* specimens; Location 1 = Edirne, 2 = Tekirdağ, 3 = Gökçeada, 4 = Bozcaada, 5 = Çanakkale (Thrace), 6 = Çanakkale (Anatolia), 7 = Marmara Island, 8 = Istanbul, 9 = Sakarya, 10 = Zonguldak, 11 = Samsun, 12 = Iğdır, 13 = Muş, 14 = Ankara, 15 = Mersin, 16 = Antalya, 17 = Manisa (Map data © Google 2020 CNES/Astrium Maxar Technologies.).

Mammalian specimens that collected and stored in the Ankara University Mammalian Research Collection (AUMAC: http://mammalia.ankara.edu.tr/) were utilized in this study. Alive specimens were captured using Sherman traps and killed by breathing diethyl ether in traps during the field studies under legal permission approved by Ankara University Local Ethics Committee for Animal Experiments (Document no: 2018-14-81). Specifically, 62 *R. rattus* from 12 locations and 37 *R. norvegicus* from eight locations (Fig. 1) were used to determine mutations in populations, properly. The time and locations for individuals being collected were given in Table S1. Species status of individuals were identified based on differences on skull, external and karyological characteristics (Yiğit et al., 2006).

The GeneAll^®^ ExgeneTM Tissue SV mini kit (Atlas Biotechnology, Ankara, Turkey) was used to isolate DNA from liver, kidney or heart tissues. PCR was performed using VKRC1ex1 primers (Forward: 5′-ATTCCTAGCTGTCACGCCTAA-3′; Reverse: 5′-CCTCCGCCAATCTTCCAATC-3′); VKRC1ex2 primers (Forward: 5′-TGGAGCTTCTT GCTAATCACTT-3′; Reverse: 5′-AGCCACGGTTACACAGAGA-3′); VKRC1ex3 primers (Forward: 5′-CCT CCT GCC TTT GCT TCT TG- 3′; Reverse: 5′ -GGA CCC ACA CAC GAT ACA CT-3′) designed with the Primer3 (v0.4.0) program (http://bioinfo.ut.ee/primer3-0.4.0/) and PCR mixtures and conditions were adapted from Yiğit, Çetintürk & Çolak (2017) optimizing primer annealing temperatures (VKRC1ex1: 60 °C, VKRC1ex2: 62 °C VKRC1ex3: 58 °C). Electrophoresis was carried out in a 0.8% agarose gel for 1 h at 70V in 1 ×TAE, and the PCR bands were visualized with a SYNGENE Bio Imaging System. Forward and reverse sequencing was performed by BMLabosis (Ankara, Turkey). Missense mutations causing amino acid changes possibly related to anticoagulant rodenticide resistance as well as silent mutations were identified with their nucleotide loci in Mutation Surveyor v5.1.2 (Minton, Flanagan & Ellard, 2011) using the ENSEMBL reference *VKORC1* sequence (ENSRNOG00000050828) of *R. norvegicus.* Sequences are available at GenBank with accession numbers MW148944 to MW149036.

## Results

### Exon 1 mutations

**Figure 2:**
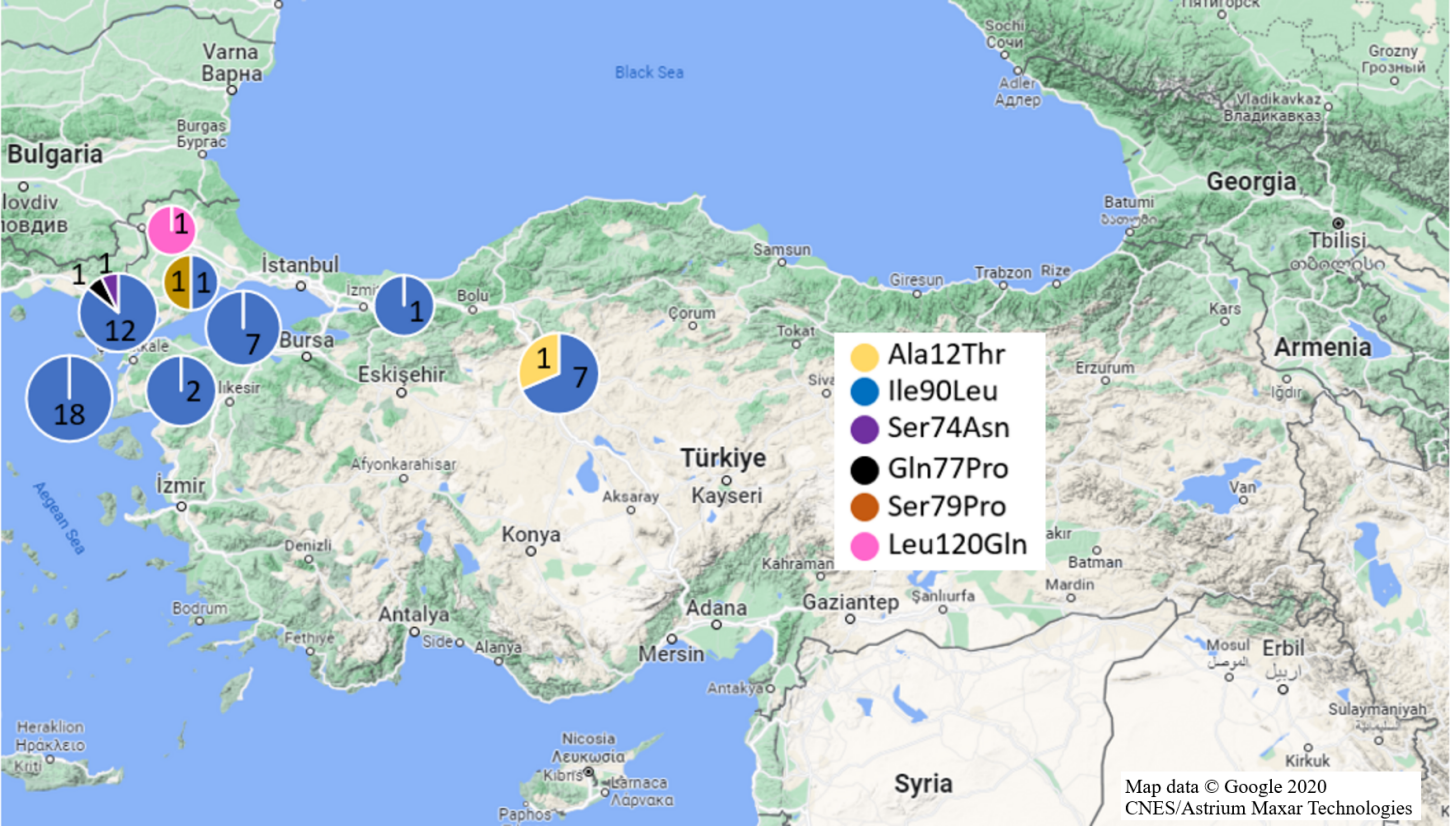
Missense mutations and distributions determined in Turkish rats. (Map data © Google 2020 CNES/Astrium Maxar Technologies).

Mutations in Exon 1 region were investigated in fifty-seven black and 36 brown rat samples (Fig. 2). As a missense mutation, only Ala21Thr (GCC>ACC) heterozygote mutation was identified in one *R. rattus* sample from Anatolia (Ankara Province). Ala21Thr mutation replace Alanine amino acid (GCC) with Threonine amino acid (ACC) in this mutation.

Silent mutations detected were Arg12Arg and Ala41Ala mutations. Whole black rat specimens (n =18/Anatolia,*n* = 39/Thrace) exhibited homozygote Arg12Arg mutations while 31.6% black rat samples had Ala41Ala mutation (homozygote: 30% Thrace; heterozygote: 100% Anatolia, 70% Thrace) (Table 1, Fig. 1). Additionally, 31.6% black rat samples (n =8/Anatolia,*n* = 10/Thrace) had both Arg12Arg and Ala41Ala mutations.

**Table 1:** Missense and silent mutations determined in Exon 1, 2 and 3 regions of *Vkorc1* gene in *R. rattus* and *R. norvegicus*.

**Exons**	**Mutations**	***R. rattus*** **(Thrace)**	***R. rattus*** **(Anatolia)**	***R. norvegicus*** **(Thrace)**	***R. norvegicus*** **(Anatolia)**
**Exon 1**	**Missense Mutations**				
	Ala21Thr (Heterozygous)	–	1	–	–
	**Silent Mutations**				
	Arg12Arg (Homozygous)	39	18	–	2
	Ala41Ala (Homozygous)	3	–	–	–
	Ala41Ala (Heterozygous)	7	8	–	1
**Exon 2**	**Missense Mutations**				
	Ile90Leu (Homozygous)	38	14	1	1
	Ser74Asn (Heterozygous)	1	–	–	–
	Gln77Pro (Heterozygous)	1	–	–	–
	Ser79Pro (Heterozygous)	–	–	1	–
	**Silent Mutations**				
	His68His (Homozygous)	36	13	6	13
	His68His (Heterozygous)	2	1	1	–
	Ser81Ser (Homozygous)	–	–	–	2
	Ile82Ile (Homozygous)	–	–	–	5
	Ile82Ile (Heterozygous)	1	–	1	5
	Leu94Leu (Homozygous)	31	14	–	–
	Leu94Leu (Heterozygous)	3	–	–	1
**Exon 3**	**Missense Mutations**				
	Leu120Gln (Heterozygous)	–	–	1	–
	**Silent Mutations**				
	Ile107Ile (Homozygous)	40	19	–	–
	Ile107Ile (Heterozygous)	–	–	–	1
	Thr137Thr (Homozygous)	39	19	–	–
	Thr137Thr (Heterozygous)	–	–	–	2
	Ala143Ala (Homozygous)	23	14	–	–
	Ala143Ala (Heterozygous)	12	3	–	2
	Gln152Gln (Heterozygous)	–	–	4	5

No missense Exon 1 mutation was detected in brown rat specimens. Homozygote Arg12Arg mutation was defined in 5.55% of brown rat samples (Ankara and Manisa provinces from Anatolia). Heterozygote Ala41Ala mutation was found in one sample from Anatolia (Manisa Province) (Table 1, Fig. 1).

### Exon 2 mutations

For the Exon 2 region, 83 samples (*n* = 55/black rat, *n* = 28/ brown rat) were used for analyses (Fig. 2). Missense Ile90Leu (ATA>TTA) mutation which transforms Isoleucine amino acid (ATA) to Leucine amino acid (TTA), was determined in 91.22% of black rat samples (*n* = 14/ Anatolia, *n* = 38/ Thrace) and 3.57% brown rat samples (*n* = 1/ Anatolia, *n* = 1/ Thrace) with 100% homozygosity. Besides this missense mutation, heterozygote Ser74Asn (Serin (AGC) to Asparagine (AAC)), and Gln77Pro (Glutamine (AAC) to Proline (CCC)) missense mutations in one Thracian black rat sample each (Edirne and Çanakkale provinces, respectively) as well as Ser79Pro (Serin (TCC) to Proline (CCC)) missense mutation in one Thracian (Tekirdağ Province) brown rat were also defined (Table 1, Fig. 1).

For black rats, His68His, Ile82Ile and Leu94Leu silent mutations were detected with a different number of homozygosity and heterozygosity (Table 1, Fig. 1). Of these specimens, 87.5% of Anatolian and 100% of Thracian samples had all His68His and Leu94Leu mutations. Only one sample from Thrace (Çanakkale Province) included Ile82Ile mutation.

In brown rats, His68His, Ser81Ser, Ile82Ile and Leu94Leu silent mutations were found. Homozygosity and heterozygosity states of these mutations were shown in Table 1 and Fig. 1. One brown rat sample (Ankara, Anatolia) had His68His, Ser81Ser and Ile82Ile mutations, one Thracian sample (Edirne Province) had both His68His and Ile82Ile mutations; the samples containing both His68His and Ile82Ile mutations are 62.5% of Anatolian and 2.56% of Thrace samples. Finally, one Anatolian sample (Ankara Province) had Leu94Leu mutation.

### Exon 3 mutations

Exon 3 region of the *Vkorc1* gene from 59 Turkish black rats and 34 brown rats were analyzed (Fig. 2). Missense mutations were not found within the studied black rats, but three silent mutations were found (Ile107Ile, Thr137Thr and Ala143Ala) in most of our sequences (88.13% of rats). Ile107Ile and Thr137Thr mutations were homozygous in all samples whereas Ala143Ala mutation differed in Anatolian and Thracian samples in terms of homozygosity and heterozygosity (Table 1, Fig. 1). In addition, approximately 10.17% black rats (50% from Turkish Thrace and 50% from Ankara) had both the Ile107Ile and Thr137Thr mutations, and one sample from Tekirdağ (Thrace) had only the Ile107Ile mutation.

The same silent mutations also appeared in brown rats at different frequencies as heterozygote; only one brown rat from Ankara (Anatolia) had all three mutations, one brown rat from Ankara (Anatolia) exhibited the Thr137Thr and Gln152Gln mutations and one brown rat Ankara (Anatolia) contained Ala143Ala mutation. On the other hand, one Thrace (Edirne Province) specimen had only Gln152Gln mutation. The heterozygous Leu120Gln (CTG>CAG) mutation relevant to anticoagulant rodenticide resistance was only found in one brown rat from Thrace (Edirne Province) (Table 1, Fig. 1). Leu120Gln mutation converted Leucine amino acid (CTG) to Glutamine amino acid (CAG).

## Discussion

While the black rat is distributed in all over Turkey with different color variations, the brown rat is more common in the humid biotopes and settlements of northern and northwestern Turkey (Yiğit et al., 1998). Anticoagulant rodenticides (ARs) are widely used for the control of rodent invasion in urban areas in Turkey like all over the world, and accordingly the mutations related to resistance was appeared in Turkish specimens despite the relatively small number of samples studied. However, mutations that cause rodenticide resistance are reported to be not occur with the same frequency in all rat populations and species (Rost et al., 2009; Grandemange et al., 2010; Haniza et al., 2013). Similarly, these mutations were not fix at high frequency in Turkish samples and also some common mutations in European samples were not detected in Turkish samples. According to the studies conducted on black rats, many missense and silent mutations have been identified around the globe (Díaz et al., 2010; Tanaka et al., 2012; Goulois et al., 2016; Cowan et al., 2017; Díaz & Kohn, 2021; Damin-Pernik et al., 2022). Likewise, many missense mutations associated with resistance to ARs have been widely reported in brown rats especially in Europe (Haniza et al., 2013; Runge et al., 2013; Meerburg et al., 2014; Boitet et al., 2017) and America (Díaz & Kohn, 2021).

Exon 1 region; Only Ala21Thr mutation was found in one black rat sample from Asiatic part of Turkey. This Ala21Thr mutation was recorded in brown rat in South Korea by Rost et al. (2009). However, it is unclear whether this mutation is related to resistance. Apart from this, Arg12Arg was detected in Turkish black rat as Cowan et al. (2017) reported in New Zealand.

Exon 2 region; Missense Ser57Phe, Trp59Arg, Trp59Cys, Arg61Trp, Phe63Cys, Glu67Lys, His68Asn, Leu76Pro and Ile90Leu and silent Ile82Ile and Leu94Leu mutations have been found in Exon 2 (Rost et al., 2009; Tanaka et al., 2012; Cowan et al., 2017; Damin-Pernik et al., 2022). Of these mutations, Ile90Leu was detected in 91.22% of black rat samples and 3.57% of brown rat specimens in Turkey, and Rost et al. (2009) regarded this mutation as neutral variant in brown rats. Cowan et al. (2017) detected this mutation in black rats from New Zealand, and also stated that black rats differ from brown rats with Ile90Ile mutation. Unlike Cowan et al. (2017), this mutation was detected in Turkish brown rats.

Cowan et al. (2017) also reported Ile82Ile and Leu94Leu mutations in Exon 2, these mutations similarly appeared in Turkish black rats. In addition, Ser74Asn, Gln77Pro (black rat) and Ser79Pro (brown rat) mutations were also found in Exon 2 in the specimens from Turkish Thrace (Table 1, Fig. 1); however, it is not clear that they could cause anticoagulant resistance and they are needed to be tested.

Exon 3 region; As a missense mutation, Leu120Gln was only detected in one brown rat sample from Turkish Thrace, this mutation was reported in brown rat from United Kingdom by Pelz et al. (2005), Buckle (2013), Vein et al. (2013), and Boitet et al. (2017). The Leu120Gln mutation has been reported to confers difenacoum, bromadiolone, warfarin and chlorophacinone resistance in brown rats (Boitet et al., 2017; Hodroge et al., 2011; Grandemange et al., 2010; Pelz et al., 2005; Haniza et al., 2013; Vein et al., 2013). Accordingly, it can be said that this mutation could also provide resistance to brown rats in Turkey. Corresponding to Cowan et al. (2017), the silent mutations of Ile107Ile, Thr137Thr and Ala143Ala in exon 3 were similarly detected in Turkish black rats, and these mutations reported in this study are likely to associated with resistance and they are required to be tested.

Regarding the resistance of ARs, most studies have focused on mutations particularly within the 139th codon in Exon 3 that confer rodenticide resistance (Pelz et al., 2005; Pelz, Rost & Müller, 2007; Lasseur et al., 2006; Rost et al., 2009; Grandemange et al., 2010; Prescott et al., 2010; Hodroge et al., 2011; Buckle, Klemann & Prescott, 2012; Buckle, 2013; Haniza et al., 2013; Runge et al., 2013; Vein et al., 2013; Meerburg et al., 2014) whereas the other mutations decrease *Vkorc1* enzyme activity (Pelz et al., 2005; Lasseur et al., 2006; Rost et al., 2009). Tyr139Phe mutation was commonly reported in French brown rats with notably high frequency of 28% (Grandemange et al., 2010). In the United Kingdom, Buckle (2013) reviewed the known missense mutations in Exon 3 as Tyr139Cys, Tyr139Phe, Tyr139Ser, Leu120Gln, and Leu128Gln from England, Tyr139Cys, Tyr139Phe, Leu120Gln and Leu128Gln from France, Leu120Gln, Tyr139Phe from Belgium, and Tyr139Cys from Germany and Denmark. Also, resistance-conferring mutations were not identified within the Irish brown rats (Mooney et al., 2018) and only one silent mutation (Ile82Ile) was observed in Exon 2 of the brown rats from New Zealand (Cowan et al., 2017). Finally, in Uganda, mutations Phe62Cys and Try139Cys were noticed (Díaz et al., 2010).

As a result; the mutations not previously associated with anticoagulant resistance may also cause the resistance, and these findings suggest that currently unidentified mutations associated with rodenticide resistance may exist in other sites within the genome of back and brown rats. Furthermore, Thracian and Anatolian populations of both rat species do not show any suitable differences in terms of mentioned mutations. The mutation type and frequency in the Turkish samples may be an evidence that the European rats with ARs resistances are not very much translocated to Turkish territory *via* shipping or highway transportation, or this may be originated due to the small sample size studied. It seems that there is no significant mutation frequency that can cause resistance. The present genetic structure probably resulted from the European-origined animal migration. In order to obtain definitive results, future studies are needed for rodenticide resistance studies to be carried out, especially in specimens with missense mutations. In any case, the results obtained will contribute to rodent control studies in Turkey.

## Conclusions

Detection of anticoagulant rodenticide resistance in rodents is of great importance for public health, rodenticide production and conservation biology. Incorrect and excessive rodenticide applications may not only be ineffective in pest control, but may also cause death of non-target organisms and excess drug accumulation in the environment. No study of *Vkorc1* mutations causing anticoagulant rodenticide resistance in Turkish rat species has yet been conducted.

In our study, missense Ala21Thr, Ile90Leu, Ser74Asn and Gln77Pro mutations in *R. rattus* specimens and Ile90Leu, Leu120Gln and Ser79Pro mutations in *R. norvegicus* specimens were determined. Only the Leu120Gln mutation is known to be related to resistance, whereas the other mutations’ relevance to resistance is unclear.

##  Supplemental Information

10.7717/peerj.15055/supp-1 Supplemental Information 1 Vkorc1 gene Exon 1 sequence file of R. norvegicus

10.7717/peerj.15055/supp-2 Supplemental Information 2 Vkorc1 gene Exon 1 sequence file of *R. rattus*

10.7717/peerj.15055/supp-3 Supplemental Information 3 Vkorc1 gene Exon 2 sequence file of *R. norvegicus*

10.7717/peerj.15055/supp-4 Supplemental Information 4 Vkorc1 gene Exon 2 sequence file of *R. rattus*

10.7717/peerj.15055/supp-5 Supplemental Information 5 Vkorc1 gene Exon 3 sequence file of *R. rattus*

10.7717/peerj.15055/supp-6 Supplemental Information 6 Vkorc1 gene Exon 3 sequence file of *R. norvegicus*

10.7717/peerj.15055/supp-7 Supplemental Information 7 Collection dates and locations of *R. rattus* and *R. norvegicus* samples used in this study

## Additional Information and Declarations

### Data Availability

The following information was supplied regarding data availability: The data is available at GenBank: MW148944 to MW149036.
