# Supplementary material for: Vkorc1 gene polymorphisms confer resistance to anticoagulant rodenticide in Turkish rats
Source: PeerJ. 2023 May 2;11:e15055. doi: 10.7717/peerj.15055 (PMC10162036; doi:10.7717/peerj.15055)
Supplement: Supplemental Information 7 [file peerj-11-15055-s007.docx]

**Supplementary Table 1.** Collection dates and locations of *R. rattus* and *R. norvegicus* samples used in this study

| Species | Collection Number | Locations with Coordinates | Collection Date |
| --- | --- | --- | --- |
| *R. rattus* | 4259 | Ankara, İskitler (39.953, 32.850) | 05.04.2022 |
| *R. rattus* | 7653 | Ankara, Atatürk Forest Farm (39.948, 32.807) | 04.07.2019 |
| *R. rattus* | 7654 | Ankara, Atatürk Forest Farm (39.948, 32.807) | 04.07.2019 |
| *R. rattus* | 7657 | Ankara, Polatlı (39.590, 32.135) | 21.07.2019 |
| *R. rattus* | 7658 | Ankara, Polatlı (39.590, 32.135) | 28.07.2019 |
| *R. rattus* | 7671 | Ankara, Polatlı (39.590, 32.135) | 9.08.2019 |
| *R. rattus* | 7672 | Ankara, Polatlı (39.590, 32.135) | 9.08.2019 |
| *R. rattus* | 4437 | Tekirdağ, Malkara (40.891, 26.904) | 7.09.2002 |
| *R. rattus* | 4478 | Çanakkale, Anafartalar (Thrace) (40.143, 26.421) | 6.11.2002 |
| *R. rattus* | 4481 | Çanakkale, Anafartalar (Thrace) (40.143, 26.421) | 11.11.2002 |
| *R. rattus* | 4482 | Çanakkale, Anafartalar (Thrace) (40.143, 26.421) | 11.11.2002 |
| *R. rattus* | 4483 | Çanakkale, Anafartalar (Thrace) (40.143, 26.421) | 12.11.2002 |
| *R. rattus* | 4485 | Çanakkale, Bigalı (Thrace) (40.236, 26.359) | 17.11.2002 |
| *R. rattus* | 4486 | Çanakkale, Bigalı (Thrace) (40.236, 26.359) | 17.11.2002 |
| *R. rattus* | 4487 | Çanakkale, Bigalı (Thrace) (40.236, 26.359) | 17.11.2002 |
| *R. rattus* | 4495 | Çanakkale, Bigalı (Thrace) (40.236, 26.359) | 26.11.2002 |
| *R. rattus* | 7132 | Çanakkale, Gelibolu (Thrace) (40.344, 26.600) | 5.08.2014 |
| *R. rattus* | 7133 | Çanakkale, Gelibolu (Thrace) (40.344, 26.600) | 5.08.2014 |
| *R. rattus* | 7134 | Çanakkale, Gelibolu (Thrace) (40.344, 26.600) | 6.08.2014 |
| *R. rattus* | 7135 | Çanakkale, Gelibolu (Thrace) (40.344, 26.600) | 6.08.2014 |
| *R. rattus* | 7136 | Çanakkale, Gelibolu (Thrace) (40.344, 26.600) | 7.08.2014 |
| *R. rattus* | 7137 | Çanakkale, Gelibolu (Thrace) (40.344, 26.600) | 7.08.2014 |
| *R. rattus* | 6996 | Çanakkale, Biga (Anatolia) (40.270, 27.405) | 20.08.2013 |
| *R. rattus* | 7254 | Çanakkale, Çan (Anatolia) (39.993, 27.077) | 24.07.2015 |
| *R. rattus* | 6822 | Marmara Island (40.625, 27.617) | 5.08.2012 |
| *R. rattus* | 6837 | Marmara Island (40.625, 27.617) | 8.08.2012 |
| *R. rattus* | 6838 | Marmara Island (40.625, 27.617) | 8.08.2012 |
| *R. rattus* | 6898 | Marmara Island (40.625, 27.617) | 16.10.2012 |
| *R. rattus* | 6899 | Marmara Island (40.625, 27.617) | 16.10.2012 |
| *R. rattus* | 6900 | Marmara Island (40.625, 27.617) | 16.10.2012 |
| *R. rattus* | 6901 | Marmara Island (40.625, 27.617) | 16.10.2012 |
| *R. rattus* | 6902 | Marmara Island (40.625, 27.617) | 16.10.2012 |
| *R. rattus* | 6908 | Marmara Island (40.625, 27.617) | 16.10.2012 |
| *R. rattus* | 7517 | Antalya, Alanya (36.498, 32.083) | 6.11.2017 |
| *R. rattus* | 7617 | Sakarya (40.765, 30.390) | 23.01.2019 |
| *R. rattus* | 6842 | Bozcaada (39.820, 26.036) | 8.08.2012 |
| *R. rattus* | 6896 | Bozcaada (39.820, 26.036) | 16.10.2012 |
| *R. rattus* | 6897 | Bozcaada (39.820, 26.036) | 16.10.2012 |
| *R. rattus* | 6903 | Bozcaada (39.820, 26.036) | 16.10.2012 |
| *R. rattus* | 6904 | Bozcaada (39.820, 26.036) | 16.10.2012 |
| *R. rattus* | 6905 | Bozcaada (39.820, 26.036) | 16.10.2012 |
| *R. rattus* | 6906 | Bozcaada (39.820, 26.036) | 16.10.2012 |
| *R. rattus* | 6907 | Bozcaada (39.820, 26.036) | 16.10.2012 |
| *R. rattus* | 6873 | Gökçeada (40.129, 25.712) | 11.09.2012 |
| *R. rattus* | 6885 | Gökçeada (40.129, 25.712) | 12.09.2012 |
| *R. rattus* | 6886 | Gökçeada (40.129, 25.712) | 12.09.2012 |
| *R. rattus* | 6887 | Gökçeada (40.129, 25.712) | 12.09.2012 |
| *R. rattus* | 6888 | Gökçeada (40.129, 25.712) | 12.09.2012 |
| *R. rattus* | 6909 | Gökçeada (40.129, 25.712) | 16.10.2012 |
| *R. rattus* | 6910 | Gökçeada (40.129, 25.712) | 16.10.2012 |
| *R. rattus* | 6911 | Gökçeada (40.129, 25.712) | 16.10.2012 |
| *R. rattus* | 6912 | Gökçeada (40.129, 25.712) | 16.10.2012 |
| *R. rattus* | 6913 | Gökçeada (40.129, 25.712) | 16.10.2012 |
| *R. rattus* | 6914 | Gökçeada (40.129, 25.712) | 16.10.2012 |
| *R. rattus* | 6939 | Mersin, Anamur (36.077, 32.833) | 12.07.2013 |
| *R. rattus* | 6953 | Mersin, Anamur (36.077, 32.833) | 15.07.2013 |
| *R. rattus* | 6954 | Mersin, Anamur (36.077, 32.833) | 16.07.2013 |
| *R. rattus* | 6955 | Mersin, Anamur (36.077, 32.833) | 17.07.2013 |
| *R. rattus* | 6969 | Mersin, Anamur (36.077, 32.833) | 31.07.2013 |
| *R. rattus* | 7056 | Manisa (38.621, 27.436) | 10.10.2013 |
| *R. rattus* | 7058 | Manisa (38.621, 27.436) | 21.10.2013 |
| *R. rattus* | 6930 | Muş, Altınova (38.684, 41.937) | 18.06.2013 |
| *R. norvegicus* | 2437 | Ankara, Maltepe (39.925, 32.844) | 1.10.1997 |
| *R. norvegicus* | 2438 | Ankara, Maltepe (39.925, 32.844) | 1.10.1997 |
| *R. norvegicus* | 2439 | Ankara, Maltepe (39.925, 32.844) | 1.10.1997 |
| *R. norvegicus* | 2440 | Ankara, Maltepe (39.925, 32.844) | 1.10.1997 |
| *R. norvegicus* | 2441 | Ankara, Maltepe (39.925, 32.844) | 1.10.1997 |
| *R. norvegicus* | 4159 | Ankara, Maltepe (39.925, 32.844) | 24.07.2001 |
| *R. norvegicus* | 4173 | Ankara, Maltepe (39.925, 32.844) | 26.07.2001 |
| *R. norvegicus* | 4237 | Ankara, Akköprü | 4.11.2001 |
| *R. norvegicus* | 4239 | Ankara, İskitler (39.953, 32.850) | 24.11.2001 |
| *R. norvegicus* | 4243 | Ankara, İskitler (39.953, 32.850) | 4.12.2001 |
| *R. norvegicus* | 4244 | Ankara, İskitler (39.953, 32.850) | 4.12.2001 |
| *R. norvegicus* | 4258 | Ankara, İskitler (39.953, 32.850) | 5.04.2002 |
| *R. norvegicus* | 4260 | Ankara, İskitler (39.953, 32.850) | 5.04.2002 |
| *R. norvegicus* | 7631 | Ankara, Çankaya (39.855, 32.828) | 23.05.2019 |
| *R. norvegicus* | 7655 | Ankara, Polatlı (39.590, 32.135) | 21.07.2019 |
| *R. norvegicus* | 7656 | Ankara, Polatlı (39.590, 32.135) | 21.07.2019 |
| *R. norvegicus* | 7721 | Ankara, Central (39.887, 32.819) | 22.08.2019 |
| *R. norvegicus* | 7724 | Ankara, Central (39.887, 32.819) | 8.11.2019 |
| *R. norvegicus* | 2794 | Samsun, Kurupelit (41.359, 36.229) | 10.09.1998 |
| *R. norvegicus* | 2808 | Samsun, Dereköy (41.461, 36.120) | 12.09.1998 |
| *R. norvegicus* | 3956 | Zonguldak, Bülent Ecevit University Campus (41.451, 31.761) | 7.12.2000 |
| *R. norvegicus* | 3957 | Zonguldak, Bülent Ecevit University Campus (41.451, 31.761) | 7.12.2000 |
| *R. norvegicus* | 3965 | Zonguldak, Bülent Ecevit University Campus (41.451, 31.761) | 1.02.2001 |
| *R. norvegicus* | 3966 | Zonguldak, Bülent Ecevit University Campus (41.451, 31.761) | 1.02.2001 |
| *R. norvegicus* | 4350 | Tekirdağ, Çerkezköy (41.291, 28.001) | 16.07.2002 |
| *R. norvegicus* | 4440 | Tekirdağ, Köseilyas Village (41.018, 27.579) | 9.09.2002 |
| *R. norvegicus* | 4392 | Iğdır (39.920, 44.044) | 7.08.2002 |
| *R. norvegicus* | 4393 | Iğdır (39.920, 44.044) | 7.08.2002 |
| *R. norvegicus* | 4394 | Iğdır (39.920, 44.044) | 8.08.2002 |
| *R. norvegicus* | 7041 | Manisa (38.621, 27.436) | 8.10.2013 |
| *R. norvegicus* | 7057 | Manisa (38.621, 27.436) | 11.10.2013 |
| *R. norvegicus* | 4430 | Edirne, Karakasım (41.516, 26.642) | 21.08.2002 |
| *R. norvegicus* | 4433 | Edirne, Karakasım (41.516, 26.642) | 23.08.2002 |
| *R. norvegicus* | 4434 | Edirne, Karakasım (41.516, 26.642) | 23.08.2002 |
| *R. norvegicus* | 4435 | Edirne, Karakasım (41.516, 26.642) | 23.08.2002 |
| *R. norvegicus* | 4438 | Edirne, İpsala (40.919, 26.380) | 7.09.2002 |
| *R. norvegicus* | 6995 | Istanbul, Silivri (41.072, 28.251) | 7.08.2013 |
